# Analgesic Drugs Combinations in the Treatment of Different Types of Pain

**DOI:** 10.1155/2012/612519

**Published:** 2012-06-11

**Authors:** Mario I. Ortiz, María Asunción Romero Molina, Young-Chang P. Arai, Carlo Luca Romanò

**Affiliations:** ^1^Laboratorio de Farmacología, Área Académica de Medicina del Instituto de Ciencias de la Salud, Universidad Autónoma del Estado de Hidalgo, Eliseo Ramírez Ulloa 400, Col. Doctores, 42090 Pachuca, HGO, Mexico; ^2^Fisiopatologia i Tratament del Dolor, IMIM, Parc de Recerca Biomèdica de Barcelona (PRBB), c/Dr. Aiguader n° 88, 08003 Barcelona, Spain; ^3^Multidisciplinary Pain Centre, School of Medicine, Aichi Medical University, Aichi 480-1195, Japan; ^4^Dipartimento di Chirurgia Ricostruttiva e delle Infezioni Osteo-Articolari, I.R.C.C.S. Istituto Ortopedico Galeazzi, Via Riccardo Galeazzi, 4, 20161 Milano, Italy

Pain relief can be achieved by a diversity of methods, with drug use being the basis of analgesic treatment. Clinical use of combinations of analgesic drugs has augmented considerably in the last few years. The purpose of combining two or more drugs with different mechanisms of action is to achieve a synergistic interaction [1], yielding a sufficient analgesic effect with lower doses, and, therefore, reduce the intensity and incidence of untoward effects. At present, many diverse classes of drugs serve as an efficient complement to nonsteroidal anti-inflammatory drugs (NSAIDs), acetaminophen or opioids, in the management of pain. But we emphasize that the success of a drug combination depends on the type of pain that is targeted (acute/chronic, inflammatory, neuropathic, cancer). Thus, opioids have frequently been used in combination with acetaminophen or NSAIDs for the clinical management of both acute and chronic pain. Likewise, the NSAIDs-acetaminophen combination has been administered to patients to relief the pain. At the end, the use of these combinations limits the doses of medication that a patient can receive. However, not all the opioid-NSAID, opioid-acetaminophen, or NSAID-acetaminophen combinations are clinically successful in all cases. For example, the association of weak opioids, such as dextropropoxyphene, to acetaminophen does not significantly increase pain relief compared to acetaminophen alone [2]. The administration of rectal acetaminophen combined with ibuprofen does not improve analgesia after adenoidectomy in the immediate postoperative period compared with either drug alone [3]. Likewise, the combination of codeine with paracetamol results in additional pain relief but may be accompanied by an increase in nausea, dizziness, vomiting, and constipation [4]. Therefore, several other combinations of analgesic agents must be evaluated experimentally or clinically to gain insight into their potential clinical use. In this sense, different combinations have been suggested. In the present special issue, a study realized by H. A. Ponce-Monter et al. showed that the diclofenac plus B vitamins combination was more effective to reduce the pain than diclofenac alone. Authors conclude that the combination of diclofenac plus B vitamins could be a safe and inexpensive postsurgical analgesic strategy. 

In the present issue, A. Porwal and coworkers showed how different drug combinations may not be equally effective in an acute pain model; in a large study population, they in fact compared diclofenac and dicyclomine injection to a combination of dexketoprofen and dicyclomine for the treatment of acute renal colic and provided evidence that the latter was significantly more effective and tolerable than the former drug combination. On the other hand, A. Hama and J. Sagen, in a comprehensive review of the available preclinical and clinical studies, illustrate the pharmacological and physiological mechanisms that justify the use of a combined drug therapy for the treatment of neuropathic pain due to spinal cord injury. The authors point out how a combination drug treatment strategy, wherein several pain-related mechanism are simultaneously engaged, may be more efficacious than treatment against individual mechanisms alone, being possible to reduce the doses of the individual drugs, thereby minimizing the potential for adverse side-effects.

Clinicians should be conscious about the benefits and risks of the drugs combination in the management of pain. Also, physicians must be aware that NSAIDs can cause potentially serious adverse effects when used in combination with other common medications such as anticoagulants, corticosteroids, or antihypertensive agents. Finally, patients should be properly counseled on the appropriate and safe use of the combination of analgesics.

*Mario I. Ortiz* *Mario I. Ortiz* *María Asunción Romero Molina* *María Asunción Romero Molina* *Young-Chang P. Arai* *Young-Chang P. Arai* *Carlo Luca Romanò* *Carlo Luca Romanò*
